# Prevalence of viral and non-viral hepatitis in Menoua Division, West Region, Cameroon: a retrospective hospital-based study

**DOI:** 10.11604/pamj.2019.32.212.16495

**Published:** 2019-04-29

**Authors:** Mathias Kenfack Tsague, Agathe Lambou Fotio, Cyrille Lionel Kamga Bomgning, Georges Nguefack-Tsague, Francois Fopa, Télesphore Benoît Nguelefack

**Affiliations:** 1 Department of Animal Biology, Faculty of Science, University of Dschang, P O Box 67 Dschang, Dschang, Cameroon; 2 Department of Zoology and Animal Physiology, Faculty of Science, University of Buea, Cameroon, PO Box 63 Buea, Buea, Cameroon; 3 Department of Public Health, Faculty of Medicine and Biomedical Sciences, University of Yaoundé I, P O Box 1364 Yaoundé, Yaoundé, Cameroon

**Keywords:** Prevalence, hepatitis B, hepatitis C, non-viral hepatitis, Menoua Division, Cameroon

## Abstract

**Introduction:**

The paucity of data on hepatitis' epidemiology in Menoua Division, west region, Cameroon, prompted us to assess the prevalence of viral and non-viral hepatitis in this area.

**Methods:**

A retrospective exhaustive study based on records of patients from January 2008 to June 2014 was conducted in 9 health centres in Menoua Division. Targeted subjects were patients who did not receive hepatitis vaccines for the past year and have been screened for hepatitis B virus (HBV), hepatitis C virus (HCV) and/or a blood transaminase. Associations between variables were quantified with odd ratios (OR) and 95% confidence interval (CI). Cochran-Armitage test of linear trend was used for testing proportions of ordinal variables. Fisher's exact test was used for testing the association between 2 qualitative variables when expected counts were less than 5.

**Results:**

The overall prevalence were 9.6% and 6.7% for HBV and HCV respectively. HBV mostly infected people aged 21-30 (12.4%) while the prevalence of HCV increased with age up to 35.4% (p=0.03). A 0.6% co-infection was observed. Thirty percent of positive HBV or HCV had high transaminase while 13% of patients with elevated transaminase showed negative viral serology.

**Conclusion:**

These results show that hospital-based prevalence of HCV and HBV in Menoua Division is under the Cameroon's national range but point out the fact that non-viral hepatitis might be a serious case of concern in this area. There is therefore, a need to identify the risk-factors of non-viral hepatitis.

## Introduction

Hepatitis is a medical condition defined as inflammation of the liver and characterized by the presence of inflammatory cells in the liver parenchyma. There are several forms of hepatitis including viral, autoimmune [1], fatty liver [2], alcoholic [3], drug-induced- [4] and toxin-induced-hepatitis. It is assumed that most cases of hepatitis are due to viral infections [5] worldwide. As such, five classes of viral hepatitis (A, B, C, D and E) are described based on their transmission routes, incubation period, and evolutionary pattern of each virus [6]. According to the World Health Organization [7] approximately 325 million people were living with chronic hepatitis infections (HBV or HCV) worldwide in 2015. It is estimated that 257 million people are living with hepatitis B virus infection while 71 million people have chronic hepatitis C infection. Africa has the highest prevalence of HCV, rated to 5.3% [8] and Cameroon is the second most afflicted with a prevalence of (4.9-13.8%) after Egypt (4.4-15.0%) [9]. Last epidemiology studies in Cameroon showed a prevalence of HBV of 13% [10]. Level of education, marital status, multiples sexual partners, scarification or tattooing, blood transfusion and surgery history have been proven to be the main risk factors associated with HBV and HCV transmission in Cameroon [11]. Xenobiotic contributes 15% to the yearly death rate due to hepatitis [12] with 2.5 million death recorded from alcoholic hepatotoxicity, representing 4% of worldwide mortality [13]. Cameroon's western region lacks critically data on hepatitis prevalence. In addition, there is no data regarding non-viral hepatitis in Cameroon, although the risk factors are prominent. Thus, this study was undertaken to assess viral (HBV, HCV) and non-viral hepatitis prevalence in Menoua Division, west region of Cameroon.

## Methods

**Figure 1:**
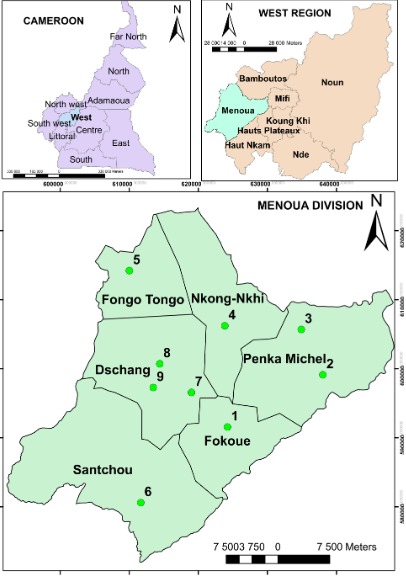
geographical localisation of Menoua Division and the nine health centres (green points) used in the present study

**Study area:** the study was conducted in Menoua Division (Figure 1), one of the 6 Divisions of the western region of Cameroon. Its population is roughly 1 million inhabitants.

**Study design:** a retrospective descriptive study was carried out to determine the evolution of the prevalence of viral hepatitis (HBsAg, anti-HCV) and non-viral hepatitis from 2008 to 2014 in Menoua Division. The hepatitis testing became available in many Menoua Subdivisions' health care centres from 2008. The nine most important health care centres of Menoua Division used in this study were: Dschang District hospital, Saint Vincent de Paul hospital, Ad Lucem hospital, Santchou Subdivision hospital, Fokoue Subdivision hospital, Fongo-Tongo Subdivision hospital, Nkong-ni CMA, Penka Michel District hospital, and Balesing CMA. Attendance registers and records from each health care centre were used. Parameters of interest were sex, age, town, serology and transaminases.

**Study population:** the study population was made up of patients attending consultation from January 2008 to June 2014 in one of the nine health centres in Menoua Division selected for the study. Targeted subjects were those who have been screened for HBV, HCV and/or have been prescribed a blood transaminase assay. Patients who had received vaccine or who had missing information were excluded from the study.

**Serological tests:** third generation Enzyme Linked Immuno-Sorbent Assay (ELISA) was used to determine the presence of hepatitis B surface antigen (HBsAg) or HCV antibodies. Patients were considered infected with HBV or HCV when they were positive to HBsAg or HCV antibodies, respectively. Blood transaminases were assayed using commercial kits, to assess liver damage. Blood transaminase values greater than 80 UI/L were considered high.

**Administrative authorisation:** to conduct this study in the different health centres, administrative authorisations were obtained from the Health District of Menoua Division and the directors of the different health centres.

**Data and statistical analysis:** qualitative variables were presented as percentages (%). Since age of patients deviated significantly from normal distribution, it was presented as median with Inter quartile range (IQR). The comparison of proportions was performed using Chi-square Test. Data were analysed using IBM-SPSS 21 (IBM SPSS Statistics for Windows, version 21 (IBM Corp., Armonk, N.Y., USA)). Associations were quantified using Odd ratios (OR) and 95% confidence interval (CI). Cochran- Armitage test of linear trend was used for testing proportions for ordinal variables. Fisher's Exact Test was used for testing association between 2 qualitative variables when expected counts were less than 5. P-values less than 5% were considered statistically significant.

## Results

**Figure 2:**
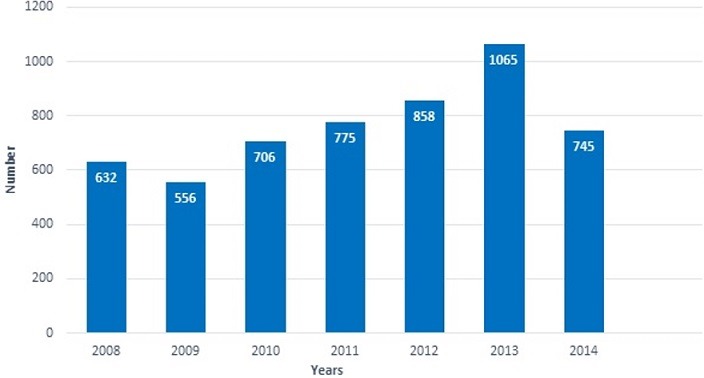
number of patients screened according to years

Among the patients screened for hepatitis or transaminases, 5337 fulfilled the inclusive conditions and did not fall under exclusive ones. The selected patients were made up of 2796 (52.4%) males and 2541 (47.6%) females with age ranging from 3 months to 101 years old with a median of 29 (IQR = 23-41 years). The number of patients screened increased significantly between 2008 and 2014 (P < 0.05) (Figure 2).

**Sex-dependent prevalence of viral hepatitis B and C:** in the tested population made up of 2239 females and 2435 males, the prevalence of HBV was 9.6%. The odd of males to be HBV infected was 1.2 times that of women (OR = 1.17 (0.97-1.41; P = 0.09)) with a prevalence of 10.3 and 8.9% respectively. The overall prevalence of HCV was 6.7% with no significant difference (p = 0.78) between male (6.6%) and female (6.9%) (Table 1).

**Table 1:** Prevalence of hepatitis B and C with respect to sex

Virus type	Sex	Negative (%)	Positive (%)	Total	OR	95%CI	P
Hepatitis B	Female	2239 (91.1)	219 (8.9)	2458	1		
	Male	2435 (89.7)	279 (10.3)	2714	1.17	0.97-1.41	0.09
	Total	4674 (90.4)	498(9.6)	5172			
Hepatitis C	Female	1590 (93.1)	117 (6.9)	1707	1		
	Male	1917 (93.4)	136 (6.6)	2053	0.75	0.75-1.25	0.78
	Total	3507(93.3)	253(6.7)	3760			

OR: odds Ratio value, CI: Confidence interval, P: P- value

**Prevalence of viral hepatitis B and C according to age:** with a prevalence of 12.4%, patients between 21-30 years were the most afflicted with HBV, followed by those aged of 31-40 (9.1%). The odds of individuals of age group 21-30 infected with HBV was 2.46 times higher than those of 0-10 (OR = 2.46 (1.19-5.09); P = 0.01). The prevalence of hepatitis C significantly increased with age (p=0.00) ranging between 2.5% to 4.7% for patients of 0-50 years. However, it significantly increases from 14.2 to 35.4% (p = 0.00) for the range 51-80. Meanwhile, the most infected groups were 61-70 years (23.8%) and 71-80 years (35.4%) (Table 2).

**Table 2:** Prevalence of hepatitis B and C with respect to age (years)

Characteristics	Negative (%)	Positive (%)	Total	OR	95%CI	P
**Hepatitis B** (P-value forCochran-Armitage test of linear trend=0.03)						
0-10	139 (94.6)	8 (5.4)	147	1		
11-20	624 (91.6)	57 (8.4)	681	1.59	0.74-3.40	0.23
21-30	1616 (87.6)	229 (12.4)	1845	2.46	1.19-5.09	0.01
31-40	868 (90.9)	87 (9.1)	955	1.74	0.82-3.68	0.14
41-50	531 (91.9)	47(8.1)	578	1.53	0.71-3.33	0.27
51-60	337 (92.3)	28 (7.7)	365	1.45	0.64-3.24	0.37
61-70	174 (93.5)	12 (6.5)	186	1.19	0.47-3.02	0.70
71 and more	98 (93.3)	7 (6.7)	105	1.24	0.43-3.53	0.68
Total	4387(90.2)	475(9.8)	4862			
**Hepatitis C** (P-value forCochran-Armitage test of linear trend=0.00)						
0-10	79 (97.5)	2 (2.5)	81	1		
11-20	400 (95.5)	19 (4.5)	419	1.87	0.42-8.21	0.40
21-30	1241(96.1)	50 (3.09)	1291	1.60	0.38-6.66	0.53
31-40	714 (95.3)	35 (4.7)	749	1.93	0.46-8.20	0.38
41-50	445 (95.3)	22 (4.7)	467	1.95	0.45-8.47	0.38
51-60	253 (85.8)	42 (14.2)	295	6.56	1.55-27.70	0.11
61-70	109 (76.2)	34 (23.8)	143	12.32	2.87-52.80	0.01
71-80	53 (64.6)	29 (35.4)	82	21.62	4.94-94.43	0.00
Total	3294(93.4)	233(6.6)	3527			

OR: odds Ratio value, CI: Confidence interval, P: P- value

**Year variations of the prevalence of viral hepatitis B and C:** the highest prevalence of hepatitis B virus was 13% in 2011, followed by 12.5 and 11.3% obtained in 2012 and 2014, respectively. The prevalence of HBV increased linearly between 2008 and 2014 (P = 0.03). The highest prevalence of HCV (9.4%) was obtained in 2009 (Table 3).

**Table 3:** Prevalence of hepatitis B and hepatitis C with respect to years

Characteristic	Negative (%)	Positive (%)	Total	OR		95%CI	P
**Hepatitis B** (P-value forCochran- Armitage test of linear trend=0.03)							
2008	589 (93.6)	40 (6.4)	629	1			
2009	509 (92.0)	44 (8.0)	553	1.27		0.82-1.99	0.27
2010	630 (91.2)	61(8.8)	691	1.42		0.94-2.16	0.09
2011	669 (87.0)	100 (13.0)	769	2.20		1.50-3.22	0.00
2012	741 (87.5)	106 (12.5)	847	2.10		1.44-3.08	0.00
2013	977 (93.0)	73 (7)	1050	1.10		0.73-1.64	0.63
2014	652 (88.7)	83 (11.3)	735	1.88		1.26-2.78	0.01
Total	4767(90.4)	507(9.6)	5274				
**Hepatitis C** (P-value forCochran- Armitage test of linear trend=0.052)							
2008	508 (92.5)	41 (7.5)	549	1			
2009	435 (90.6)	45 (9.4)	480	1.28		0.82-1.99	0.01
2010	486 (91.7)	44 (8.3)	530	1.12		0.72-1.75	0.27
2011	484 (96.8)	16 (3.2)	500	0.41		0.22-0.74	0.62
2012	587 (94.7)	33 (5.3)	620	0.70		0.44-1.11	0.01
2013	629 (92.8)	49 (7.2)	678	0.96		0.63-1.49	0.13
2014	427(94.1)	27 (5.9)	454	0.79		0.47-1.30	0.88
Total	3556(93.3)	255(6.7)	3811				

OR: odds Ratio value, CI: Confidence interval, P: P- value

**Prevalence of viral hepatitis B and C according to Sub-division:** out of the 6 Sub-divisions, Santchou and Dschang were the most affected with an overall prevalence of 10.5 and 9.9% respectively for HBV, while the highest prevalence of HCV was in Santchou (13.3%) (Table 4).

**Table 4:** Prevalence of hepatitis B and hepatitis C with respect to sub-division

Characteristics	Negative (%)	Positive (%)	Total	OR	95%CI	P
**Hepatitis B** (P-value for Fisher's Exact Test=0.11)						
Dschang	4179 (90.1)	457 (9.9)	4636	1		
Nkong-Ni	191 (95.0)	10 (5.0)	201	0.48	0.25-0.91	0.02
Penka Michel	363 (91.0)	36 (9.0)	399	0.90	0.63-1.30	0.60
Santchou	34 (89.5)	4 (10.5)	38	1.08	0.39-3.05	0.90
Total	4767 (90.4)	507 (9.6)	5274			
**Hepatitis C** (P-value for Fisher's Exact Test=0.03)						
Dschang	3386 (93.1)	249 (6.9)	3635	1		
Penka -Michel	157 (97.5)	4 (2.5)	161	0.35	1.12-1.95	0.38
Santchou	13 (86.7)	2 (13.3)	15	2.09	1.47-9.32	0.33
Total	3556 (93.3)	255 (6.7)	3811			

OR: odds Ratio value, CI: Confidence interval, P: P- value

**Overall results of viral hepatitis B and C in the studied population:** among the patients screened for both HBV and HCV, there were 0.6% co-infection. Among the 5337 patients included in the study, 86.0% neither had HBV nor HCV, while 13.4% either had (not both) HBV or HCV.

**Association between serological test and transaminase rate:** out of the 5337 patients who underwent the viral serology tests, only 132 were screened for the level of transaminases. Thirty percent of the 132 patients had high transaminase (> 80 UI) level linked to either HBV or HCV positive serology. Among the HBV and HCV co-infected patients, 20% had elevated level of transaminases. The last group (13%) showed a negative serology with a high level of transaminase (Table 5).

**Table 5:** Association between serological test and rate of transaminase

	Both HVB- and HVC- n (%)	Either HVB+ or HVC+ n (%)	Both HVB+ and HVC+ n (%)	Total n (%)
**High level ALAT** (P-value for Fisher's Exact Test=0.17)				
No	64 (83.1)	20 (66.7)	4 (80.0)	88 (78.6)
Yes	13 (16.9)	10 (33.3)	1 (20.0)	24 (21.4)
Total	77 (100.0)	30(100.0)	5 (100.0)	112 (100.0)
**High level ASAT** (P-value for Fisher's Exact Test=0.02)				
No	63(81.8)	19 (63.3)	2 (40.0)	84 (75.0)
Yes	14(18.2)	11 (36.7)	3 (60.0)	28 (25.0)
Total	77(100.0)	30(100.0)	5(100.0)	112 (100.0)
**Either (not both) ALAT high or ASAT high** (P-value for Fisher's Exact Test=0.11)				
No	60 (89.5)	18 (85.7)	2 (50.0)	80 (86.9)
Yes	7 (10.5)	3 (4.3)	2 (50.0)	12 (13.1)
Total	67(100)	21(100.0)	4(100.0)	92 (100.0)
**High level of both ALAT and ASAT** (P-value for Fisher's Exact Test=0.05)				
No	60 (85.7)	18 (66.7)	2 (66.7)	80 (80.0)
Yes	10 (14.3)	9 (33.3)	1(33.3)	20 (20.0)
Total	70 (100.0)	27 (100.0)	3 (100.0)	100 (100.0)

ALAT: alanine aminotransferase, ASAT: aspartate aminotransferase, HVB: viral hepatitis B, HVC: viral hepatitis C

## Discussion

This study aimed to assess the prevalence of viral and non-viral hepatitis in Menoua Division. It was found that the number of patients screened significantly increased with time between 2008 and 2014. This shows the increasing awareness of hepatitis in the general population. Furthermore, governmental policy has improved the availability of diagnostic tools which could also explain the increasing number of patients screened. Out of 5337 patients screened, 9.6% and 6.7% were infected respectively with HBV and HCV. The HBV prevalence of 9.6% obtained here is similar to the 9.2% recorded by Njouom [14] in the western region, but lower than the general prevalence in Cameroon (13%). Nevertheless, Menoua Division is still a region of concern. The high prevalence of HBV in the general population of Cameroon may be justified by the fact that screening, vaccination and treatment are by individual approaches [15]. Furthermore, the cost of the vaccination and screening test is still high for an average Cameroonian. Concerning HCV, several countries like Bolivia, Burundi, Cameroon, Egypt, Guinea, Mongolia, Rwanda and Tanzania have a prevalence above 10% [16], Cameroon being the second most affected after Egypt [9]. Results from the present study demonstrated that the prevalence of HCV in the Menoua Division is 6.7%. Although this prevalence is less than the general' observed in Cameroon (11.6%), it is higher than the 4.8% obtained by Noubiap *et al*. [17] in blood donors population in Edea, Cameroon. The HCV prevalence results corroborates with the 7.7% reported by Mbanya [18] among pregnant women in Yaoundé, Cameroon. It is worth noting that in Cameroon, HCV prevalence varies from simple to more than double from one region to another. Regional prevalence is therefore of a paramount importance in order to design management strategies against the disease. Hepatitis B is more prevalent (13.8%) than hepatitis C (11.6%) in the general population of Cameroon [14] notwithstanding the prominent campaign of hepatitis B vaccine. Those findings are in line with results obtained in the present study and further indicate that there is still more to do in order to control the transmission of the disease. Particular attention needs to be paid on medical interventions, sexual activities and disease transmission from mother to child [19]. It was also noteworthy that until the end of 2014, it was still not possible to do HBV and HCV serological testing in Fokoué and Fongo-tongo Sub-divisions. This demonstrates the insufficiency of health care policy orientated to hepatitis in Cameroon, despite the efforts made by the government.

Santchou Sub-division was the most affected by hepatitis B and hepatitis C, followed by Dschang Sub-division. The high prevalence observed in Santchou might likely be tributary to limited knowledge of this disease by its rural population mainly made up of farmers. In addition, Santchou is a cosmopolitan area compared to other Sub-divisions. According to Blachier *et al*. [20], patients infected with hepatitis live in the high majority in peripheral areas of Africa and Asia where sensitization, screening and access to treatment is not easily available. The high prevalence observed in Dschang could also be explained by the high number of students arriving each year. Our results showed that men were mostly infected by HBV while women were the most infected by HCV. These results contrast with those got by Arifa [5] in three Pakistan Districts. Nevertheless, they are similar to those obtained by Ankouane [21] who checked the prevalence of hepatitis B in a population of bank employees in Cameroon according to age, the range 21-30 years was the most screened and showed the highest prevalence of HBV. This could be explained by the fact that young people in Cameroon usually get married at that age; moreover, some clinical examinations including hepatitis checking especially are part of their prenuptial preparations. These results agree with those previously obtained [14, 15, 18, 22]. The prevalence of HCV highly increased with age and the most affected people were those of 71-80 years (35.4%). Similar results were obtained by Pepin *et al*. [11] in Ebolowa where the prevalence was 56% in age group ≥ 60 years old. Thirty percent of patients who have underwent both the transaminase and viral serology test had high transaminase (> 80 UI) level associated to either HBV or HCV while 13% showed a negative serology with a high level of transaminase. This result indicates that viruses are not the sole cause of liver damage or liver disease. Non-viral factors may surely be highly involved. Megarbane [12] reported that 15% of hepatitis is caused by xenobiotics. Indeed, alcoholism is a source of liver injury, and lead to 2.5 million deaths each year [13]. Furthermore, William [23] reported that drug-induced hepatic injury is the most frequent reason and accounts for more than 50 percent of the cases of acute liver failure in the United States. The use of some chemical reagents like carbon tetrachloride also produce liver damage [24, 25]. There are many other viruses bringing about hepatitis including cytomegalovirus, epstein-barr virus, yellow fever virus, mump virus and rubella virus [26] which may also account for this liver injury. Nevertheless, since Agriculture is the main activity in Menoua Division, huge quantities of pesticides is used without proper care; this could be another cause of liver damage. Whatever the case, it clearly appears that non-viral hepatitis is a serious case of concern in Menoua Division.

## Conclusion

The prevalence of viral hepatitis in Menoua Division is high though lower than the national rate. The HBV is more prominent than HCV but there is no difference as far as sex is concerned. Santchou Sub-division was the most affected and people of 20-29 years are the most affected. The prevalence of non-viral hepatitis is also high in this region.

**Limits and recommendations:** hepatitis management policy should consider both viral and non-viral types, especially in the Menoua division; update epidemiological data of infectious and non-infectious hepatitis at regional and national levels to assist decision-making; identify the risk-factors of non-viral hepatitis; sensitise the local population about the transmission of hepatitis and most importantly, on the precaution to avoid non-viral hepatitis.

### What is known about this topic

- Cameroon is one of the countries with higher hepatitis prevalence;
- The general prevalence of HBV in Cameroon is about 13%;
- Africa has the highest prevalence of HCV, estimated to 5.3%.

### What this study adds

- This article presents for the first time, the prevalence of hepatitis in the Menoua Division (West Cameroon), and rise the case of non-viral hepatitis in the studied region;
- The paper helps to understand that the causes of hepatitis in Menoua Division are not only viral. Indeed, the prevalence of non-viral hepatitis is higher than that of viral hepatitis.

## Competing interests

The authors declare no competing interests.
